# Associations between the use of herbal medicines and adverse pregnancy outcomes in rural Malawi: a secondary analysis of randomised controlled trial data

**DOI:** 10.1186/s12906-018-2203-z

**Published:** 2018-05-25

**Authors:** Collins Zamawe, Carina King, Hannah Maria Jennings, Edward Fottrell

**Affiliations:** University College London, Faculty of Population Health Sciences, Institute for Global Health, 30 Guildford Street, London, WC1N 1EH UK

**Keywords:** Herbal medicines, Medicinal plants, Pregnant women, Labour, Pregnancy outcomes, Pregnancy complications, Neonatal death, Neonatal morbidity

## Abstract

**Background:**

The use of herbal medicines during pregnancy is very high globally and previous studies have pointed out possible associations with adverse pregnancy outcomes. Nevertheless, the safety of herbal medicines in pregnancy is under-explored in low-income countries experiencing high maternal and neonatal complications. We investigated the associations between self-reported use of Mwanamphepo (a group of herbal medicines commonly used to induce or hasten labour) and adverse maternal and neonatal outcomes in rural Malawi.

**Methods:**

We conducted a cross-sectional analysis of secondary household data relating to 8219 births that occurred between 2005 and 2010 in Mchinji district, Malawi. The data were collected as part of a cluster-randomised controlled trial (RCT) that evaluated community interventions designed to reduce maternal and neonatal mortality. Data were gathered on maternity history, demographic characteristics, pregnancy outcomes and exposure to Mwanamphepo. Associations between self-reported use of Mwanamphepo and maternal morbidity as well as neonatal death or morbidity were examined using mixed-effects models, adjusted for relevant covariates. All analyses were also adjusted for the clustered nature of the survey.

**Results:**

Of the 8219 births, Mwanamphepo was used in 2113 pregnancies, representing an estimated prevalence of 25.7%. The self-reported use of Mwanamphepo was significantly associated with increased occurrence of maternal morbidity and neonatal death or morbidity. Specifically, the odds of maternal morbidity were 28% higher among self-reported users than non-users of Mwanamphepo (AOR = 1.28; 95% CI = 1.09–1.50) and the probabilities of neonatal death or morbidity were 22% higher (AOR =1.22; 95% CI = 1.06–1.40) among neonates whose mother reportedly used Mwanamphepo than those who did not.

**Conclusion:**

The use of Mwanamphepo was associated with adverse pregnancy outcomes in rural Malawi. Thus, herbal medicines may not be safe in pregnancy. Where possible, pregnant women should be discouraged from using herbal medicines of unconfirmed safety and those who report to have used should be closely monitored by health professionals. The study was limited by the self-report of exposure and unavailability of data relating to some possible confounders.

## Background

Herbal medicine is a healing approach based on the use of plants or plant extracts [1, 2], and is one of the most common complementary and alternative therapies in pregnancy around the world. Though methods vary, studies have shown that up to 80% of pregnant women in Italy [3], 55% in the UK [4], 40% in Palestine [5], 35% in Taiwan [6] and 50% in Zimbabwe [7] utilised herbal remedies between 2009 and 2015. The indications for herbal medicines differs from place to place and can be clinical or non-clinical. The frequently reported clinical indications for herbal medicine use are nausea and vomiting, labour pain, induction of labour, swollen feet and back pain [8–11]. On the other hand, the non-clinical motives include poor access to health facilities, cultural beliefs and practices associated with pregnancy, dissatisfaction with biomedical health systems and the belief that herbal remedies are relatively safe and effective [12–14].

Although herbal medicines are considered comparatively safe by some pregnant women, this claim is not based on evidence and may be incorrect for two reasons. First, owing to lack of sufficient data on safety, the risk of adverse effects associated with herbal medicine use during pregnancy may be higher compared to conventional medicines [15, 16]. Secondly, the safety of any drug, including herbs, cannot be guaranteed in pregnancy because of the possible teratogenic effects [3, 17, 18]. So far, there is no agreement on the safety of herbal medicines among pregnant women in the literature [19, 20]. Some studies have demonstrated that herbal medicines are safe during pregnancy [21–23], whereas others have shown that they contain active substances that may be harmful to both the woman and the foetus [24–26]. This raises many questions regarding the safety of herbal medicines during pregnancy that need to be addressed. Considering the high use of herbal medicines during pregnancy [7, 27] and poor maternal and neonatal outcomes [28, 29] in many low-income countries, it is important to explore the possible link between the two in such settings yet studies on herbal medicine’s safety have been mostly restricted to high and upper middle-income countries.

Malawi is one of the poorest countries in the world, with a maternal mortality ratio (MMR) of 439 deaths per 100,000 live births and a neonatal mortality rate (NMR) of 27 deaths per 1000 births [30]. The prevalence of herbal medicine use is yet to be assessed in the country; however, anecdotal evidence suggests that utilisation during pregnancy is common [31, 32]. For instance, a study in a rural Mulanje district found heavy reliance on plants for medicine and documented over 20 plant species that are frequently used during pregnancy [31]. The possible relation between the use of herbal medicines and adverse pregnancy outcomes in Malawi was first noted four decades ago after a maternal death survey discovered that a significant number of cases were diagnosed with toxic effects of herbs [33]. A few years later, Bullough and Leary [34] conducted a follow-up animal study and found that some of the herbal medicines that traditional birth attendants (TBA) prescribed to pregnant women had oxytocic properties. They argued that the use of such medicines while under the care of unskilled birth attendants could be harmful to pregnant women. This has been corroborated by a recent national maternal death review commissioned by the Malawi Ministry of Health, which found that some of the traditional medicines that women used during pregnancy induced strong uterine contractions that often result in maternal morbidity and death [35].

Mwanamphepo (*cissus/vitaceae plants species*) is a local Malawian name used to describe a group of herbal medicines that are commonly used by pregnant women to induce or hasten labour [31, 32]. It is often mixed with porridge and taken orally by pregnant women who are due for delivery. To date, there are no published studies that have assessed associations between Mwanamphepo use and adverse pregnancy outcomes. Given that Malawi did not make sufficient progress towards reducing maternal mortality in the last 15 years [29], it is critical to understand if the use of herbal medicines is one of the stumbling blocks. This could provide evidence necessary for policies and interventions concerning the use of herbal medicines and other alternative therapies during pregnancy. To this end, this study was undertaken to describe the prevalence of and factors associated with Mwanamphepo use and evaluate the associations between self-reported use of Mwanamphepo and adverse maternal and neonatal outcomes in rural Malawi.

## Methods

### Study design and setting

We conducted a cross-sectional analysis of household survey data that were collected between 2005 and 2010 as part of a Cluster-Randomised Controlled Trial (RCT) intended to evaluate women’s groups and volunteer counselling interventions in Mchinji District, Malawi. The RCT, including the study interventions, is described in detail elsewhere [36–38].

### The source of data and population

During the RCT, Mchinji district was divided into 48 clusters of about equal-sized population and 12 of these were controls [37]. All women of childbearing age (WCBA) who fell pregnant in the 48 clusters were interviewed at one-month and six-months after childbirth by trained interviewers to retrospectively gather data about maternity history, care seeking practices, including use of Mwanamphepo, and pregnancy outcomes [36]. In the present study, we only included data collected one-month after delivery due to substantial missing data and potential recall biases in the follow-up (6 months) interviews. Moreover, we only used data from the 12 control clusters to minimise the possible effects of the RCT interventions on herbal medicine use and pregnancy outcomes.

### Conceptual framework

**Fig. 1:**
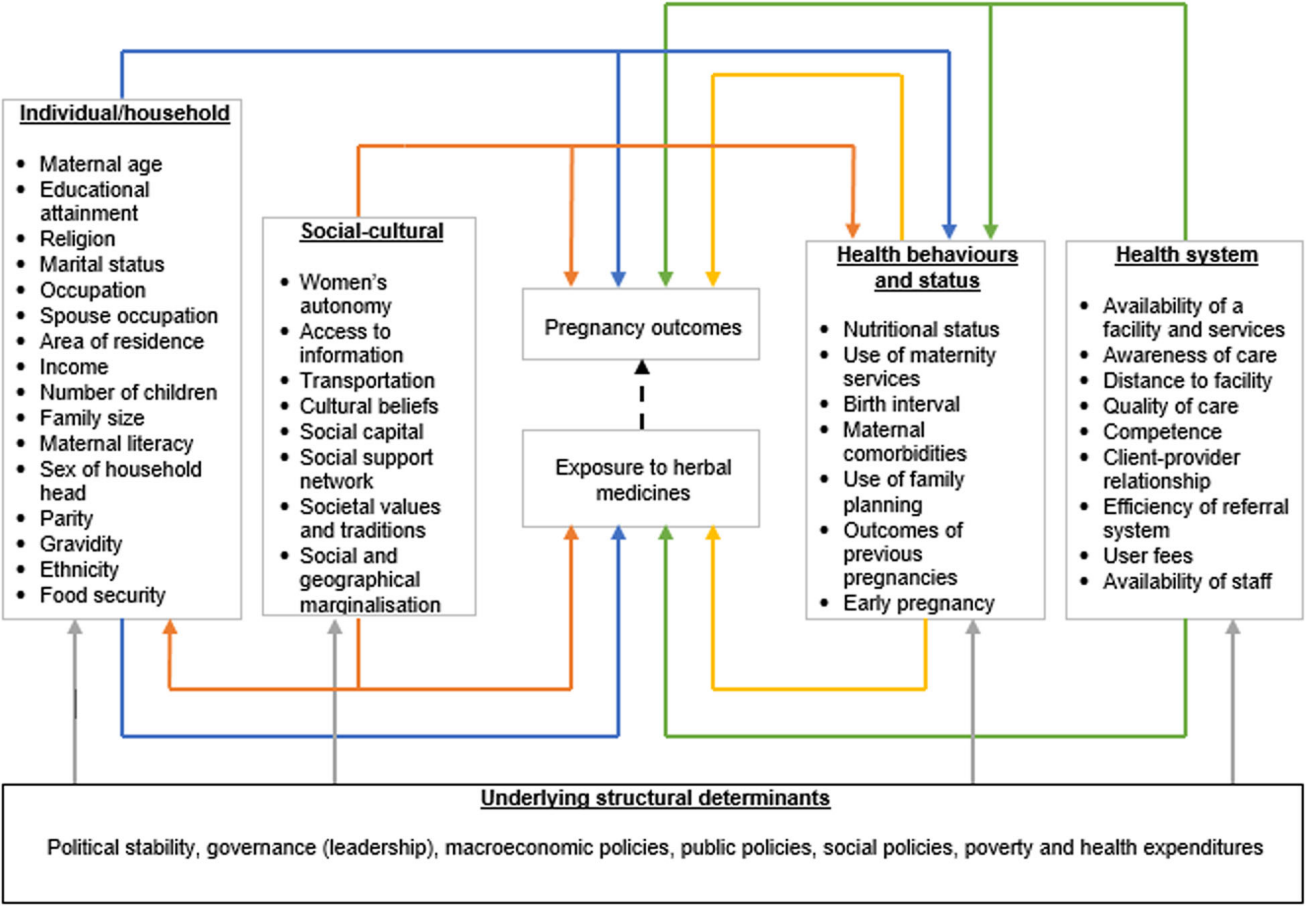
Hypothetical determinants of pregnancy outcomes and the use of herbal medicines among pregnant women. The relationship between exposure to herbal medicines and pregnancy outcomes is not clearly understood, hence, the broken line

To guide our analysis, we identified and mapped conceptual determinants of both pregnancy outcomes and the use of herbal medicines during pregnancy as shown in Fig. 1. The theoretical framework has been adapted from a World Health Organisation’s discussion paper [39] and a 10-year review of literature by Stokoe [40], which discuss the determinants of maternal mortality in the developing world. It is also based on the works of Stanifer et al. [41] in Tanzania, Laelago et al. [42] in Ethiopia and Nyeko et al. [43] in Uganda about factors associated with the use of herbal medicines in pregnancy. The framework permits us to hypothesise the relationship between herbal medicine use and pregnancy outcomes in limited resource settings as well as explore potential confounders that needs to be accounted for in the analysis.

### Variables and measurements

The self-reported use of Mwanamphepo is the exposure of interest (independent variable) in this study and the outcome measures (dependent variables) are self-reported maternal morbidity and neonatal death/morbidity. The maternal morbidity variable combines the following outcomes: caesarean section, assisted vaginal delivery, premature rupture of membrane, any postnatal morbidity and any delivery problem. The neonatal death/morbidity variable combines neonatal death, meconium-stained liquor, low birth weight, preterm birth and any neonatal morbidity. Covariates were included based on their theoretical relevance to the topic [40, 41] and this was informed by the conceptual framework (Fig. 1). The variables that were identified and included in all adjusted analyses are: attendance of antenatal clinic, attendance of postnatal clinic, place of delivery, number of antenatal visits, timing of the first antenatal visit (gestational age), age, marital status, tribe, occupation, wealth tertiles and religion. Wealth tertiles were constructed through principal components analysis involving the following variables: type of floor, type of roofing, owns land, source of water, employed a household worker, persons per household room, electricity at home, and ownership of motorbike, oxcart, car, bicycle and radio.

### Data management and analysis

As part of the data quality control in the original study, births and deaths identified by enumerators were independently confirmed by an interviewer and a random sample of the interviews were repeated by field supervisors. Completed questionnaires were checked in full for completeness and consistency by a team of data checkers. Further reviews were done in the databases after data entry to check for discrepancies and missing data. Mistakes were addressed by checking the questionnaires or consulting the interviewers or call-backs to the field for verification.

In the present analysis, original variables were recoded or combined to suit the purpose of this study and checked for consistency, errors, missing data and outliers. The level of missing data on all variables was very low (< 5%), hence cases with missing values were only excluded from the analyses that included them (analysis by analysis basis - pairwise deletion) [44]. As data were clustered at individual (e.g. some women had more than one pregnancy) and trial cluster levels, mixed-effects models and *svyset* (Stata command) were used to adjust for clustering. The main analysis involved comparing maternal and neonatal adverse outcomes among users and non-users of Mwanamphepo through mixed-effects models. For each outcome measure, crude and adjusted models were specified and all analyses were performed using Stata/SE 13.1 (StataCorp LP, Texas, USA). The significance level of 5% and 95% confidence interval were used.

### Ethics statement

The original RCT was granted ethical permission in Malawi by the National Health Sciences Research Committee (MED/4/36/I/167) and in the UK by UCL Institute of Child Health and Great Ormond Street Hospital [36]. In the original study, verbal informed consent was obtained from each participant and this procedure was duly approved by the ethics committees. Verbal consent was considered as appropriate due to a high level of illiteracy in the study setting.

## Results

A total of 8286 births were recorded in the 12 clusters and data on Mwanamphepo use was obtained for 8219 births representing a response rate of 99.2%. Of the 8219 births, Mwanamphepo was used in 2113 pregnancies, representing a prevalence of 25.7% (Table 1).

**Table 1:** Characteristics of participants and factors associated with utilisation of Mwanamphepo during pregnancy (Mixed-effects model, adjusted for clustering)

Characteristics	Total		No herbal remedies utilised		Utilised herbal remedies		COR (95% CI) *p*-value	^a^AOR (95% CI) *p*-value
	*n*	% or m(SD)	*n*	% or m(SD)	*n*	% or m(SD)		
	8219	100%	6106	74.3%	2113	25.7%		
Age (years)	7834	26.9 (6.7)	5823	27.3 (6.7)	2011	25.8 (6.7)	0.96 (0.95–0.97) < 0.001	0.96 (0.95–0.98) < 0.001
Highest level of education								
None (Ref.)	2440	29.7%	1828	30.0%	611	28.9%		
Primary	5313	64.7%	3899	63.9%	1414	67.0%	1.06 (0.92–1.23) 0.439	0.95 (0.80–1.14) 0.601
Secondary or higher	457	5.6%	370	6.1%	87	4.1%	0.68(0.50–0.93) 0.018	0.68 (0.47–0.99) 0.042
Marital status								
Currently married (Ref)	6858	83.5%	5205	85.3%	1653	78.3%		
Never married	1012	12.3%	646	10.6%	366	17.3%	1.80 (1.49–2.18) < 0.001	1.30 (0.93–1.82) 0.118
Formerly married^b^	343	4.2%	250	4.1%	93	4.4%	1.21 (0.82–1.53) 0.474	1.10 (0.76–1.58) 0.615
Main occupation								
Farming (Ref)	6993	85.2%	5250	86.1%	1743	82.6%		
Salaried employee	128	1.6%	100	1.6%	28	1.3%	0.68 (0.40–1.15) 0.150	0.98 (0.53–1.81) 0.942
Self employed	210	2.5%	178	2.9%	32	1.5%	0.57 (0.36–0.90) 0.016	0.75 (0.45–1.26) 0.281
Student/no work	878	10.7%	570	9.4%	308	14.6%	1.73 (1.42–2.11) < 0.001	1.25 (0.88–1.77) 0.216
Wealth tertiles								
Lowest (Ref)	2753	34.1%	1967	32.6%	786	38.3%		
Middle	2655	32.8%	1962	32.5%	693	33.7%	0.91 (0.77–1.06) 0.216	0.97 (0.81–1.16) 0.759
Highest	2673	33.1%	2099	34.8%	574	28.0%	0.68 (0.57–0.80) < 0.001	0.83 (0.69–0.99) 0.053
Religion								
Christian (Ref)	7965	97.0%	5920	97.1%	2045	96.9%		
Muslim and others	244	3.0%	179	2.9%	65	3.1%	0.78 (0.54–1.13) 0.185	1.07 (0.69–1.66) 0.768
Ethnicity								
Chewa (Ref)	7886	96.1%	5867	96.2%	2019	95.7%		
Ngoni	199	2.4%	135	2.2%	64	3.0%	1.11 (0.74–1.65) 0.615	0.98 (0.62–1.55) 0.925
Others	125	1.5%	97	1.6%	20	1.3%	0.82 (0.48–1.41) 0.476	0.76 (0.39–1.45) 0.403
Attended antenatal clinic (ANC)								
No (Ref)	337	4.1	269	4.4%	68	3.2%		
Yes	7869	95.9	5826	95.6%	2043	96.7%	0.83 (0.59–1.17) 0.286	1.50 (0.35–6.35) 0.582
Gestational age (month) at first ANC	7572	5.8 (1.3)	5617	5.8 (1.3)	1995	5.7 (1.2)	0.92 (0.92–1.03) 0.305	0.29 (0.90–1.03) 0.276
Unsuccessful last pregnancy								
No (Ref)	6172	95.1%	4731	95.5%	1441	93.9%		
Yes	319	4.9%	225	4.5%	94	6.1%	1.33 (1.13–1.54) 0.002	1.32 (1.12–1.54) 0.001
At least four ANC visits								
No (Ref)	4982	65.8%	3609	66.1%	1274	65.2%		
Yes	2586	34.2%	1905	33.9%	689	34.8%	0.97 (0.84–1.12) 0.687	0.97 (0.81–1.16) 0.731
Place of delivery								
Hospital/health facility (Ref)	3662	46.2%	3004	51.1%	658	32.3%		
Traditional birth attendants	2191	27.7%	1414	24.0%	777	38.1%	3.71 (3.07–4.47) < 0.001	3.65 (2.94–4.54) < 0.001
Home/on the way to facility	2068	26.1%	1465	24.9%	603	29.6%	2.13 (1.79–2.59) < 0.001	2.31 (1.87–2.83) < 0.001
Received postnatal care								
No (Ref)	4144	58.4%	3024	57.9%	1120	59.7%		
Yes	2953	41.6%	2198	42.9%	755	40.3%	0.87 (0.75–1.01) 0.064	1.18 (1.00–1.39) 0.059

*COR* Crude odds ratio, *AOR* Adjusted odds ratio, *SD* Standard deviation, *M* mean, *CI* confidence interval

^a^Adjusted for attendance of antenatal clinic, attendance of postnatal clinic, place of delivery, number of antenatal visits, timing of the first antenatal visit (gestational age), age, marital status, tribe, occupation, wealth tertiles and religion

^b^Includes widowed, divorced and separated

Women who delivered with a traditional birth attendant (AOR = 3.65; 95% CI = 2.94–4.54) and at home or on the way to a health facility (AOR = 2.31; 95% CI = 1.87–2.83) were more likely to utilise Mwanamphepo than those who delivered in a health facility. The odds of Mwanamphepo use were 17% lower among women in the highest wealth tertile than their counterparts in the lowest (AOR = 0.83; 95% CI = 0.69–0.99). Unsuccessful previous pregnancy (e.g. stillbirth, neonatal death) was associated with the use of Mwanamphepo in the current pregnancy (AOR = 1.32; 95% CI = 1.12–1.54). Increasing age was significantly associated with decreased likelihoods of using Mwanamphepo (AOR = 0.96; 95% CI = 0.95–0.98). Characteristics of the participants and other factors related to the utilisation of Mwanamphepo during pregnancy are provided in Table 1.

The self-reported use of Mwanamphepo was significantly associated with increased odds of both maternal morbidity and neonatal death or morbidity. In particular, the odds of maternal morbidity were 28% higher among users than non-users of Mwanamphepo (AOR = 1.28; 95% CI = 1.09–1.50). The odds of neonatal death or morbidity were 22% higher among neonates whose mothers reportedly used Mwanamphepo than those who did not (AOR =1.22; 95% CI = 1.06–1.40). The strength of the relationship between maternal morbidity and Mwanamphepo slightly increased after adjusting for covariates whereas that of neonatal death/morbidity and Mwanamphepo attenuated but remained significant (Table 2).

**Table 2:** Associations between exposure to Mwanamphepo during pregnancy and adverse pregnancy outcomes (Mixed-effects model, adjusted for clustering)

Outcome measures	Total n (%)	Non-exposed n (%)	Exposed n (%)	COR (95% CI) p-value	^a^AOR (95% CI) p-value
Maternal morbidity					
No (Rc)	6100 (74.2%)	4665 (76.4%)	1435 (67.9%)		
Yes	2119 (25.8%)	1441 (23.6%)	678 (32.1%)	1.21 (1.05–1.38) 0.006	1.28 (1.09–1.50) 0.002
Neonatal death or morbidity					
No (Rc)	3538 (43.1%)	2795 (45.8%)	744 (35.2%)		
Yes	4680 (56.9%)	3311 (54.2%)	1369 (64.8%)	1.30 (1.16–1.46) < 0.001	1.22 (1.06–1.40) 0.006

*COR* Crude odds ratio, *AOR* Adjusted odds ratio, *CI* Confidence interval, *Rc* Reference category

^a^Adjusted for attendance of antenatal clinic, attendance of postnatal clinic, place of delivery, number of antenatal visits, timing of the first antenatal visit (gestational age), age, marital status, tribe, occupation, wealth tertiles and religion

## Discussion

The self-reported use of Mwanamphepo was associated with adverse pregnancy outcomes in rural Malawi. The users tended to experience more pregnancy-related complications and the probabilities of death/morbidity was higher for their neonates compared to non-users. Thus, there is evidence to suggest a hypothesis that the use of Mwanamphepo during pregnancy is a risk factor for maternal and neonatal complications. In the literature, there is conflicting evidence regarding this proposition. While some studies have shown that the use of herbal medicines during pregnancy is associated with adverse pregnancy outcomes [24, 25, 45, 46], others did not find such evidence [19, 45, 47]. This discrepancy could be due to variations in or lack of data on types of herbal medicines used, dosages or length of exposure, time of use (seasonality or stage of pregnancy) and study locations among others [48, 49]. This underlines the need for rigorous assessment of exposures in studies evaluating the safety of herbal medicines.

The prevalence of self-reported use of Mwanamphepo among pregnant women in Mchinji district was 25.7%. As far as we know, this is the first study to estimate the prevalence of herbal medicine use in Malawi and thus it will serve as a benchmark for future studies in the country. The prevalence is however, considerably different from those observed in many sub Saharan countries [7, 24, 42, 50]. For instance, studies in South Africa, Zimbabwe, Ethiopia and Nigeria have all reported estimates greater than 50% [7, 24, 42, 50]. There are two possible explanations for this inconsistency. First, the use of herbal medicines is culturally determined [12, 43, 51]; hence, the lower prevalence of use observed in the present study could be attributed to the differences in cultural settings. Secondly, the focus of this study was relatively narrow as it was particularly interested in the use of Mwanamphepo and for that reason other herbal medicines used during pregnancy were possibly not reported. Notwithstanding the lack of agreement with other studies, we strongly believe that our prevalence estimate is more reliable and representative of the population under study due to relatively large sample size and coverage area.

Giving birth at home or with a TBA appears to be related to the use of Mwanamphepo and this is not surprising as TBAs are known for prescribing herbal medicines [35, 52, 53]. We have found that an unsuccessful previous pregnancy increased the possibility of using Mwanamphepo. In sub Saharan Africa, women are responsible for protecting the pregnancy from spiritual harm and those who do not, may be blamed if the pregnancy fails [51, 54]. So, if a woman lost her previous pregnancy, it is reasonable to suggest that she would want to take extra precautions by among others turning to herbal medicines [55]. It has been noted that higher household wealth, secondary education and an increase in maternal age were associated with decreased probabilities that a pregnant woman would utilise Mwanamphepo. Generally, there is little to no consensus on the link between demographic characteristics and herbal medicine use in pregnancy as studies have reported conflicting evidence [7, 42, 43, 56, 57]. On maternal age, young women could be more worried about the pregnancy than older women due to lack of experience and this could motivate them to fully obey traditional practices, including the use of herbal medicines, but this is likely to change over time (or with age).

The study had limitations and strengths worth acknowledging. Since both exposure and outcomes data were self-reported by the participants as well as collected at the same time, recall and social desirability biases cannot be ruled out. The data used in this study was collected for a different purpose, as such the exposure of interest may not have been rigorously measured. For example, the dosage of mwanamphepo and actual time of exposure were not recorded. Data were collected on Mwanamphepo only and because of this it was not possible to adjust for the impact of other herbal medicines that may have been utilised. The data may also be outdated as the RCT started in 2005. The strength of the study lies in its large sample size and extended fieldwork, which suggest robust statistical significance.

## Conclusion

Herbal medicines may not be safe during pregnancy. In this study, we have established that the use of Mwanamphepo during pregnancy in rural Malawi was associated with adverse pregnancy outcomes. Although we are unable to draw a causal-effect relationship due to the nature of our study, this observation cannot be taken lightly, especially in countries experiencing high maternal and neonatal mortality as well as morbidity. We call upon health professionals to familiarise themselves with the herbal medicines commonly utilised by pregnant women in their work settings and deliberately create a conducive environment that could facilitate disclosure of herbal medicine use during consultations. As a precaution, those who admit having used herbal medicines should be closely monitored. Where possible, pregnant women should also be discouraged from using herbal medicines of unverified safety. Although the current study has gone some way towards enhancing our understanding of the safety of Mwanamphepo during pregnancy, further work needs to be undertaken to confirm if the use of Mwanamphepo or other types of herbal medicine is a risk factor for pregnancy complications.

## Abbreviations

AOR: Adjusted odds ratio
CI: Confidence interval
ILO: Induction of labour
MMR: Maternal mortality ratio
NMR: Neonatal mortality rate
RCT: Randomised controlled trial
SD: Standard deviation
TBA: Traditional birth attendants
WCBA: Women of child bearing age
WHO: World health organisation
